# Utilizing BMP-2 muteins for treatment of multiple myeloma

**DOI:** 10.1371/journal.pone.0174884

**Published:** 2017-05-10

**Authors:** Axel Seher, Charlotte Lagler, Thorsten Stühmer, Urs Dietmar Achim Müller-Richter, Alexander Christian Kübler, Walter Sebald, Thomas Dieter Müller, Joachim Nickel

**Affiliations:** 1 Department of Oral and Maxillofacial Plastic Surgery, University Hospital Würzburg, Würzburg, Germany; 2 Department of Internal Medicine II, University Hospital Würzburg, Würzburg, Germany; 3 Department Physiological Chemistry II, Theodor-Boveri-Institute (Biocentre), University of Würzburg, Würzburg, Germany; 4 Julius-von-Sachs-Institute, Department Molecular Plant Physiology and Biophysics, University of Würzburg, Würzburg, Germany; 5 Chair Tissue Engineering and Regenerative Medicine, University Hospital Würzburg, Würzburg, Germany; 6 Fraunhofer IGB, Translational Centre Würzburg "Regenerative Therapies in Oncology and Musculoskeletal Diseases", Würzburg, Germany; Universite de Nantes, FRANCE

## Abstract

Multiple myeloma (MM) represents a haematological cancer characterized by the pathological hyper proliferation of antibody-producing B-lymphocytes. Patients typically suffer from kidney malfunction and skeletal disorders. In the context of MM, the transforming growth factor β (TGFβ) member Activin A was recently identified as a promoter of both accompanying symptoms. Because studies have shown that bone morphogenetic protein (BMP)-2-mediated activities are counteracted by Activin A, we analysed whether BMP2, which also binds to the Activin A receptors ActRII and ActRIIB but activates the alternative SMAD-1/5/8 pathway, can be used to antagonize Activin A activities, such as in the context of MM. Therefore three BMP2 derivatives were generated with modified binding activities for the type II (ActRIIB) and/or type I receptor (BMPRIA) showing either increased or decreased BMP2 activity. In the context of MM these BMP2 muteins show two functionalities since they act as a) an anti-proliferative/apoptotic agent against neoplastic B-cells, b) as a bone-formation promoting growth factor. The molecular basis of both activities was shown in two different cellular models to clearly rely on the properties of the investigated BMP2 muteins to compete for the binding of Activin A to the Activin type II receptors. The experimental outcome suggests new therapeutic strategies using BMP2 variants in the treatment of MM-related pathologies.

## Introduction

Multiple myeloma (MM) is a malignant disease of bone marrow characterized by a pathological increase in antibody-producing plasma cells and is thus inherently linked to an accompanying increase in immunoglobulins (plasmacytosis) [1]. The pathological concomitant phenomena of this disease are hypercalcaemia, increased susceptibility to infections and organ malfunction, which are caused by the deposition of antibody fragments. Patients also suffer from the extremely painful destruction of bone structure [2]. The incidence of MM is approximately 4–6 new cases per 100,000 people per year. MM represents 10% of all haematological and 1% of all cancer types [3]. To date, the exact mechanisms leading to the manifestation of this disease are not well understood, but are probably not monocausal [4,5].

Recently, Activin A, a member of the TGFβ superfamily, has come to the forefront as a new attractive target for novel therapeutic strategies in the field of MM. Activin A might cause the two most prominent symptoms associated with MM: enhanced plasma cell proliferation and bone osteolysis or osteonecrosis. In 2010, Vallet *et al*. published that the degree of osteolysis in patients with MM correlates first with elevated Activin A levels in the blood plasma and second with a pronounced inhibition in osteoblast differentiation [6]. *In vivo* experiments using the adoptive transfer of human myeloma cells into mice demonstrated that a synthetic decoy against Activin A, a soluble Activin A type II receptor termed RAP-011, not only led to increased osteoblast activity but also limited the growth of neoplastic B-cells and significantly improved overall bone integrity [6]. Similar results were reported by Chantry *et al*. using a similar Activin A decoy receptor (ActRIIA.muFc) in a murine myeloma model. In these studies, treatment with ActRIIA.muFc resulted in the stimulation of osteoblastogenesis and inhibition of the myeloma-induced suppression of bone formation. Furthermore, ActRIIA.muFc blocked the formation of osteolytic bone lesions and increased animal survival rates [7]. Sotatercept, the human analogue of ActRIIA.muFc, has since been successfully tested in phase II clinical trials. MM patients treated with Sotatercept showed decreased osteolytic lesions and attenuated tumour activity [8]. This result is in excellent agreement with previous findings that reported a correlation between increased Activin A blood serum levels and the degree of bone lesions [9].

The biological role of Activin A in the context of these pathologies has been described well. The TGFβ ligand Activin A not only inhibits osteoblast differentiation [10] but also activates bone-resorbing osteoclasts and thereby exerts a strong catabolic effect in bone tissue homeostasis. Additionally, haematopoietic bone marrow cells, which undergo differentiation into osteoclasts upon stimulation with receptor activator of NF-κB ligand (RANKL) and macrophage colony-stimulating factor (M-CSF), showed increased expression in the RANKL receptor RANK when treated with Activin A. Therefore, increasing the susceptibility of these cells to RANKL will result in the amplification of osteoclast differentiation via a forward-powered paracrine/autocrine loop [11].

However, the precise molecular mechanism regarding how Activin A antagonists might promote the osteoanabolic effects as well as the reduction in cancer cell proliferation in the context of MM has not been revealed or clearly understood. In 2016 Aykul et al. described a ligand-mediated mechanism for signaling regulation of the type II TGFβ family receptors ActRII, ActRIIB, and BMPRII, that can explain the antagonistic effect of Activin A on a molecular level. As Activin A and bone growth-promoting BMPs not only share but also utilize highly overlapping epitopes at these type II receptors they hypothesized a direct competition mechanism as the basis of mutual antagonism. Hereby, ligands that bind type II receptors with high affinity, like Activin A, effectively compete with ligands that interact with type II receptors with low affinity, like BMP2, BMP7 and BMP9, for binding to the type II receptor and thereby block BMP signaling. They also proposed that such a signaling antagonism is an integral function of the TGFβ signal transduction system [12]. This postulated antagonism presents a very good model to explain the effects observed for Activin A antagonists in MM. In this work, we show that Activin A directly inhibits BMP2-induced anti-proliferative/apoptotic activities in MM cell lines. To counteract Activin A activity we engineered BMP2 variants which exhibit increased receptor binding affinities for particular type II receptors and can thus not only be utilized as superior Activin A antagonists, but simultaneously serve as BMP2 super-agonists to facilitate bone growth. These features might be clinically exploited as a new therapeutic option for the treatment of MM.

## Materials and methods

### Cell culture

The human MM cell lines MM.1S, RPMI8226, AMO1, U266, L363, JJN3, OPM2, KMS12-BM, KMS11 and INA6 cells were cultured in RPMI 1640 (PAA, Pasching, Germany) supplemented with 10% (v/v) heat-inactivated foetal bovine serum (PAA, Pasching, Germany). Recombinant human IL6 (ImmunoTools, Friesoythe, Germany) was added to a final concentration of 2.ng/ml when culturing INA6 cells. All assays with MM cell lines were carried out with 10% FCS in RPMI and for the INA6 cells also 2 ng/ml IL6 was added. The cell lines MM.1S, RPMI 8226 und U266 were purchased from the American Type Culture Collection (ATCC). The cell lines C2C12, KMS12-BM, AMO1, OPM2, L363, JJN3 were obtained from the Leibniz-Institut DSMZ (Deutsche Sammlung von Mikroorganismen und Zellkulturen GmbH), Braunschweig, Germany. The cell line ATDC5 was purchased from Sigma-Aldrich, Darmstadt, Germany. The cell line INA-6 was a friendly gift from Dr. Martin Gramatzki, Erlangen.

### Ligand expression

Recombinant human BMP2, including the variants BMP2-L51P (termed BMP2-P), BMP2-L100KN102D (termed BMP2-KD) and BMP2-L51PL100KN102D (termed BMP2-PKD), were expressed in *E*. *coli*, refolded and purified as previously described [13]. Recombinant human Activin A was produced in baculovirus-transfected SF9 insect cells as described [14].

### WST-1 assay

Cell proliferation was analysed using the WST-1 reagent according to the manufacturer's recommendations (Roche, Germany). Briefly, 20,000 cells/well were seeded in 96-well plates. The cells were then incubated for 72 h in the presence or absence of recombinant ligands. Ten microliters of WST-1 (Roche, Germany) was added per well, and the cells were further incubated for 2–3 h at 37°C. CD138-positive primary cells were incubated for 5 to 7 h with WST-1. The concentration of formazan generated was determined at 450 nm wavelength using an ELISA plate reader. The figures show the mean values of triplicates and the standard deviation. Assays were conducted as three independent experiments.

### Alkaline phosphatase (ALP) assay

The murine pre-chondrogenic cell line ATDC5 (RIKEN, no. RCB0565) was cultivated in DMEM/Ham’s F12 containing 5% FCS and antibiotics (100 U ml^-1^ penicillin G and 100 μg ml^-1^ streptomycin). The murine myoblast cell line C2C12 (ATCC CRL-1772) was maintained in DMEM containing 10% FCS. The ALP assays were performed in the ATDC5 or C2C12 cells as previously described [15]. The figures show the mean values and the standard deviation of triplicates. The assays were carried out as three independent experiments.

### Quantitative reverse-transcription PCR (qRT-PCR)

The following primers were used for the qRT-PCR experiments:

1. ActRI (forward) TACGATGTGGTTCCCAATGA and (reverse) AGTCTTGCGGATGGATTTTG;
2. ActRIB (forward) CGACTTAGTGCCCTCTGACC and (reverse) TGTGGAGAGAGGGAGCAGTT;
3. ActRII (forward) GTTGCCATTTGAGGAGGAAA and (reverse) CCAGCTGATAACCTGGCTTC;
4. ActRIIB (forward) CTGACTTTGGCTTGGCTGTT and (reverse) AGGGCAGCATGTACTCATCC; and
5. HPRT (hypoxanthine-guanine phosphoribosyltransferase) (forward) GACCAGTCAACAGGGGACAT and (reverse) ACACTTCGTGGGGTCCTTTT.

Total RNA was prepared using the Qiagen RNeasy Mini Kit (Qiagen, Hilden, Germany) according to the manufacturer´s recommendations. For cDNA synthesis, 1 μg of total RNA was reverse-transcribed using the QuantiTect Reverse Transcription Kit (Qiagen, Germany). qRT-PCR was performed using 20 ng of the cDNA synthesis mix per reaction and the QuantiTect SYBR Green PCR Kit (Qiagen, Hilden, Germany). Three independent PCR analyses were performed in duplicate for each gene. Relative expression levels were calculated from a comparison with the house keeping gene HPRT and the following equation: rel. Expression (%) = [2^(CtS-CtR)^]*100, where CtS is the Ct value for HPRT gene expression and CtR is the Ct value for the individual receptor gene expression.

### Primary MM cells/CD138^+^ selection

Bone marrow aspirates from MM patients were obtained at the Universitätsklinikum Würzburg, Medizinische Klinik und Poliklinik II after obtaining informed consent. Permission was granted by the local ethics committee (Ethik-Kommission der Medizinischen Fakultät der Universität Würzburg; reference number 18/09). The mononuclear cell fraction was isolated via density centrifugation (Lymphocyte Separation Medium; PAA) and rinsed with phosphate-buffered saline (PBS). After a second wash with cold separation buffer (PBS containing 0.5% FBS and 2.5 mmol/l EDTA), the cell suspension was incubated for 15 min at 8°C with CD138 Microbeads (Miltenyi Biotech, Bergisch Gladbach, Germany) in a rotating shaker. CD138-positive cells were then isolated using MACS Large Cell Columns (Miltenyi Biotech), spun and resuspended in complete medium (10% FBS) supplemented with 2 ng/ml IL6. Cells were seeded at a density of 30,000 cells/well into 96-well plates, and Activin A (125 nM) was either added or not. Cell purity was proofed as described before [16]. The cells were subsequently cultured for 3 days prior to the WST assay, and the final values were calculated relative to the untreated control cells. Each assay was performed in duplicate unless low MM cell numbers permitted only a single measurement.

## Results

**Fig 1:**
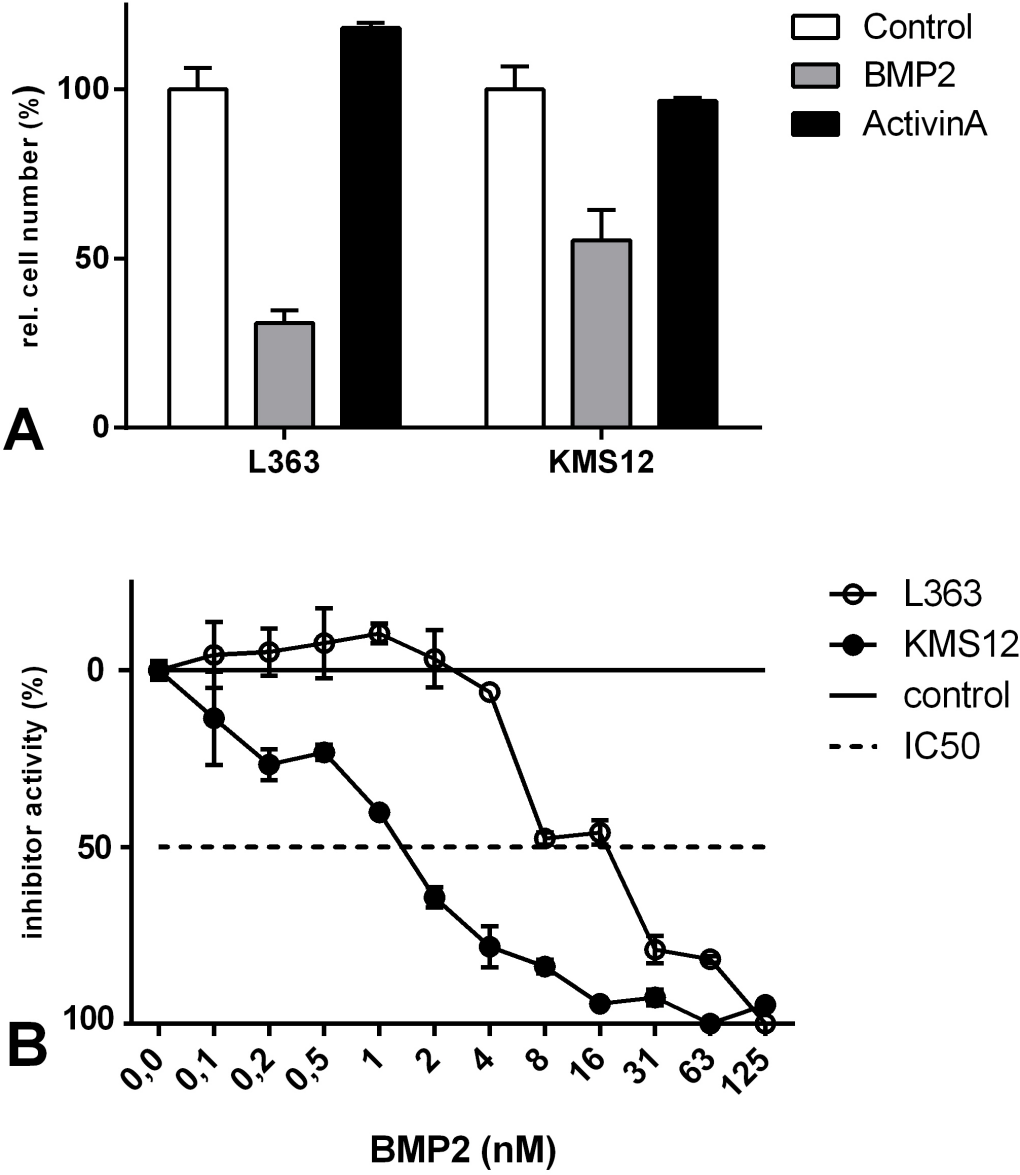
BMP2, but not Activin A, downregulates relative cell number in the L363 and KMS12-BM myeloma cell lines. **(A)** Cells were grown in the presence or absence of 125 nM BMP2 or Activin A for 72 h. Relative cell numbers were assessed by WST-1 measurements. (B) For a detailed analysis the dose response curve of BMP2 was determined for L363 (black circle line) and KMS12-BM (black circle) after incubation with BMP2 for 72h via WST1 assay. To allow comparison of the IC50 values, the dose-response curves were normalized with the value measured in the absence of BMP2 set to zero. The figures show the mean values and standard deviation of triplicates. The assays were carried out as three independent experiments.

BMP2 was originally identified as a promoting factor for the development of bone tissue [17,18], but it also acts as an anti-proliferative and/or apoptotic factor on MM cells. Aside from BMP2, BMP4, -5, -6, -7 and -9 have also been described to similarly exert such anti-proliferative/apoptotic functions in different human MM cell lines as well as in human primary MM cells [19–23]. Because Activin A has also been designated as an apoptotic factor in MM cell lines [24], we first investigated the effect of BMP2 and Activin A on the proliferation of selected MM cell lines by measuring relative cell numbers. Exposure to 125 nM BMP2 significantly attenuated cell proliferation up to 70% in L363 cells and to 45% in KMS12-BM cells. In contrast, exposure to the same concentration of Activin A did not significantly affect proliferation (Fig 1A). For a detailed analysis the dose-dependency of BMP2-mediated inhibition of proliferation was determined for both cell lines (Fig 1B).

**Fig 2:**
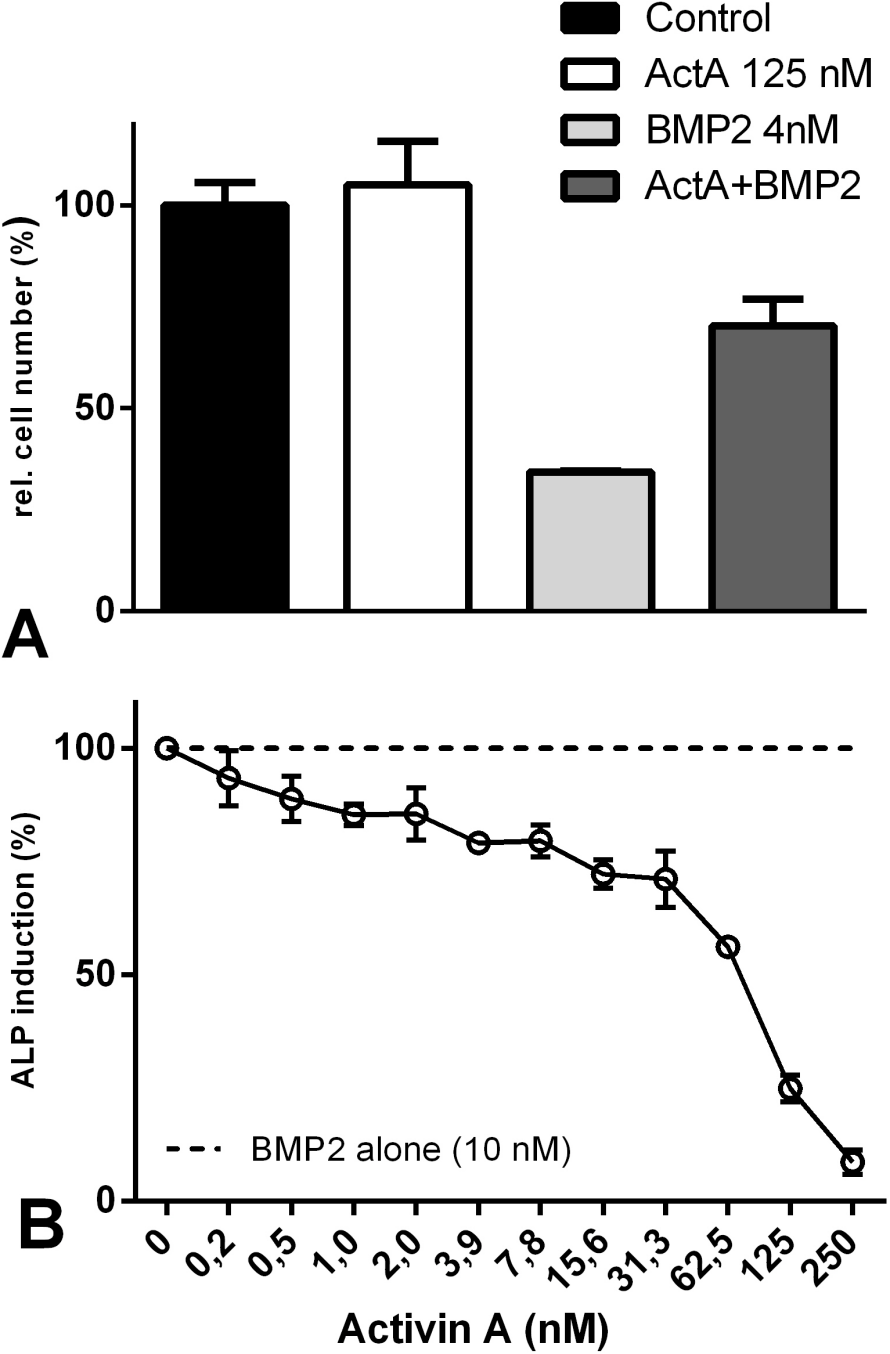
Activin A inhibits BMP2-mediated cellular responses. (A) KMS12-BM cells were stimulated with either 125 nM Activin A or 4 nM BMP2 alone or co-stimulated with both growth factors for 72 h. Cell growth was assessed by WST-1 measurements. (B) C2C12 cells were incubated without BMP2, 10 nM BMP2 and 10 nM BMP2 plus increasing concentrations of Activin A. After 72 h, ALP activity was measured by determining p-nitrophenylphosphate conversion using an ELISA reader. In the absence of BMP2 cells showed no ALP activity and the background signal was set as 0%. ALP activity derived from stimulation with 10 nM BMP2 was defined as 100% (dashed line). Addition of Activin A without BMP2 yielded no ALP activity (data not shown). The figures show the mean values and the standard deviation of triplicates. The assays were performed as three independent experiments.

Next, we investigated whether Activin A antagonizes the BMP2-mediated anti-proliferative effects in KMS12-BM cells. Therefore, we incubated the cells simultaneously with BMP2 (4 nM) and Activin A (125 nM). The presence of Activin A significantly reduced the anti-proliferative effects mediated by BMP2 (Fig 2A, fourth bar). A similar antagonism was observed when analysing alkaline phosphatase (ALP) expression—a marker for osteogenic differentiation—in the C2C12 pre-myoblast cell line. In the non-stimulated cell line ALP is not expressed. The addition of BMP2 induced ALP gene expression, which could be almost completely inhibited by simultaneously adding Activin A (Fig 2B). A similar inhibitory effect of Activin A on ALP expression has been reported for BMP7 [25].

Thus, Activin A and BMP2 appear to form a mutually antagonizing pair of growth factors. In principal, mutual antagonism can be achieved by two different mechanisms: either the two antagonizing factors activate two distinct signalling cascades that encode for opposing activities, or both factors bind to one or more shared receptor components through which different biological activities are generated. The latter mechanism is an assumed to be a key feature of TGFβ superfamily members, as most members bind promiscuously to overlapping sets of BMP receptors. To initiate signal transduction, TGFβ ligands bind and assemble two type I and two type II transmembrane receptors into active heteromeric signalling complexes [26]. Because more than 30 ligands have to pair with only seven type I and five type II receptor chains, a pronounced promiscuity exists for the interactions between ligands and receptors [26]. Furthermore the signal appears to converge even further, as canonical signalling is limited to only two principal pathways, the SMAD1/5/8 or SMAD2/3 pathways. Which of these two pathways will be activated appears to depend solely on the type I receptor utilized in the active ligand-receptor complex [26,27]. BMP2 and Activin A recruit different type I receptors. BMP2 signals via BMPRIA or BMPRIB and hence activates the SMAD1/5/8 pathway. In contrast, Activin A activates the SMAD2/3 pathway by engaging ActRIB in its ligand-receptor complex. Due to the activation of different SMAD pathways, the observed mutual antagonism might therefore result from genetically encoded opposing functions. However on the other hand, both ligands utilize the same type II receptors, ActRII and ActRIIB, thereby creating a competitive binding situation for BMP2 and Activin A, which could also account for the counteracting properties. In such a scenario, Activin A potentially acts as a BMP2 antagonist and vice versa. This process would depend on the presence of the particular type II receptors on the target cells and quantitatively depend on the local concentrations of the individual ligands. In this case, the efficiency of the mutual antagonism also strongly depends on the affinities of the two ligands to the shared receptor.

We tested our hypothesis of direct competition via mutual antagonism by using BMP2 variants with specifically altered type II receptor binding characteristics using a set of previously designed BMP2 variants enabling us to address defined receptor complexes with distinct signalling capacities [28]. The double amino acid mutation L100K and N102D in BMP2 yielded a variant (BMP2-KD) that binds ActRII (3.5-fold) and ActRIIB (20-fold) with significantly increased affinities, while binding to BMPRII is unchanged. The elevated affinities of this BMP2 variant for both Activin type II receptors should result in an enhanced antagonizing capacity against Activin A, as the latter shares the Activin type II receptors with BMP2.

**Fig 3:**
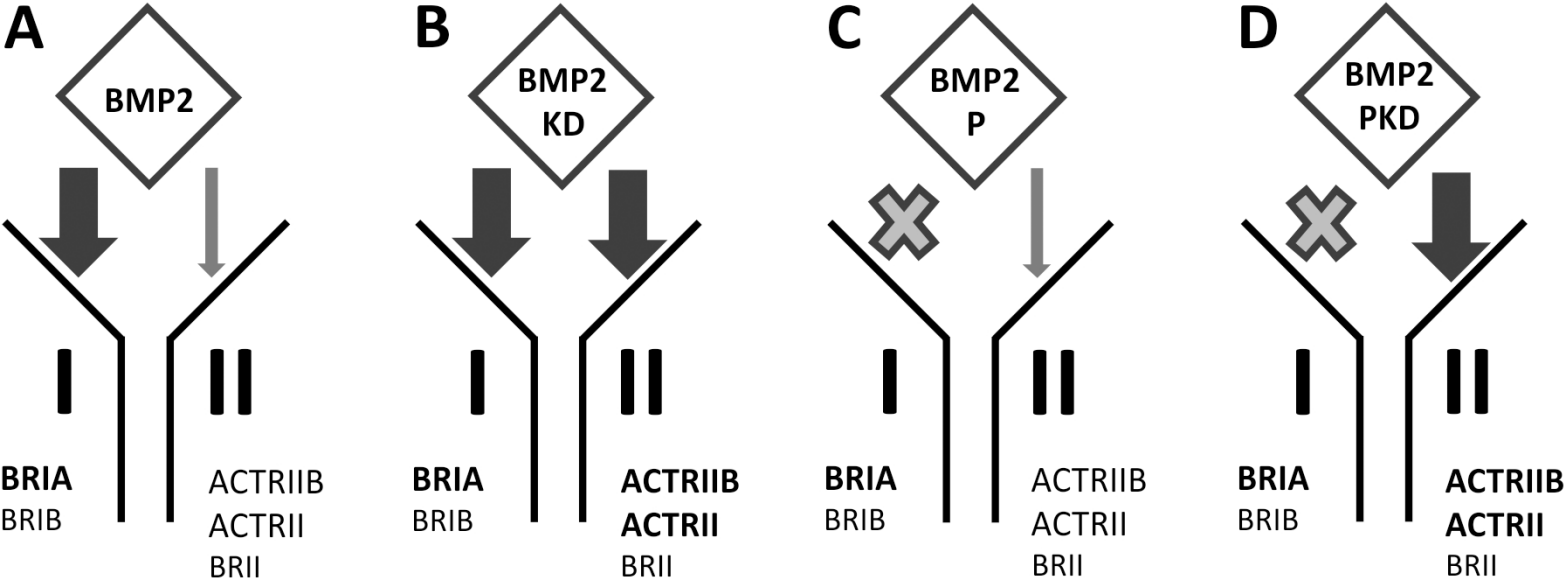
Schematic representation of the BMP2 variants in terms of their receptor binding characteristics. The arrows demonstrate the relative binding affinities of the individual BMP2 variants to the indicated receptors. High-affinity binding is indicated by thick black arrows, while low-affinity binding is indicated by thin grey arrows. For both “P” variants, there was no binding (indicated by the grey cross) to the type I receptors BRIA and BRIB that could be detected by surface plasmon resonance (SPR) analysis [15,28]. Bold receptor names indicate binding preferences among the two receptor types.

**Fig 4:**
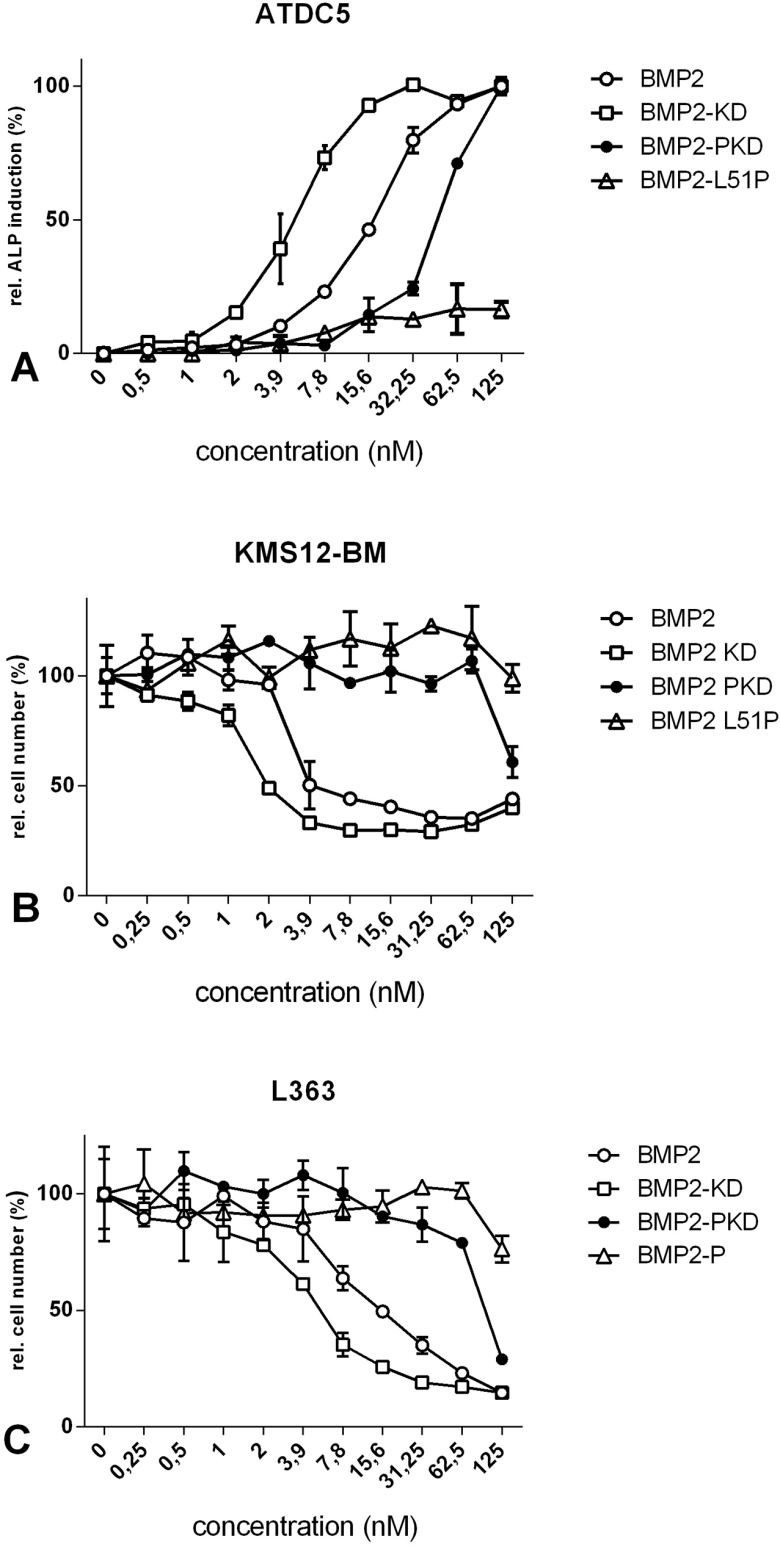
Biological activities of the different BMP2 variants. (A) ATDC5 cells were stimulated with the indicated concentrations of either wildtype BMP2, BMP2-KD, BMP2-PKD or BMP2-P. ALP activity was determined after 72 h. (B) KMS12-BM and (C) L363 cells were stimulated with the indicated concentrations of the four BMP2 variants. Inhibition of proliferation was assessed after 96 h by WST-1 measurements. The figures show mean values and standard deviation of triplicates. The measurements were performed in three independent experiments.

To rule out BMP2-mediated changes in gene expression as the cause of Activin A antagonism, we introduced a third mutation. We previously described a BMP2 variant that has a strongly reduced binding affinity for the type I receptors BMPRIA or BMPRIB. This variant was generated by exchanging leucine at position 51 for proline. Leucine 51 was identified as a hot spot for type I receptor binding in BMP2 and although the resulting protein (BMP2-P) was termed to be "type I receptor-dead" by Keller and co-workers since it does not exhibit any BMP signaling capacity, this BMP2 variant retains all binding characteristics to BMP2-specific type II receptors as well as to BMP-antagonizing modulator proteins such as NOGGIN [29]. The combination of the above-mentioned mutations yielded the variant BMP2-PKD, which binds Activin type II receptors with enhanced affinities compared to wildtype BMP2, but like BMP2-P, does bind to BMP2 type I receptors with very low affinities. To better understand and illustrate the different signalling/binding capacities, the BMP2 variants are depicted in Fig 3. While BMP2-P cannot initiate any BMP signalling, the variant BMP2-PKD exhibits strongly attenuate BMP signaling (Fig 4A).

To determine the bioactivities of these mutants, we analysed their osteoanabolic potential by measuring the induction of ALP expression in ATDC5 cells (Fig 4A). As reported earlier, wildtype BMP2 induces ALP activity in these cells in a dose-dependent manner yielding an EC50 value of approximately 7 nM. The EC50 values for the induction of ALP by wildtype BMP2 determined in our study are in excellent agreement with published data [30]. The BMP2-KD variant exhibited a slightly increased activity as deduced from an approximately two-fold lower EC50 value. However, both BMP2 mutants, BMP2-P and BMP2-PKD carrying the L51P mutation showed either no or a significantly reduced ALP induction compared with wildtype BMP2 or the variant BMP2-KD.

We next addressed the potential bioactivities of these variants in the context of MM. KMS12-BM (Fig 4B) and L363 (Fig 4C) cells were exposed to either ligand, and their effects on cell proliferation were analysed. Our results clearly show that both wildtype BMP2 as well as the variant BMP2-KD significantly reduced cell proliferation in a dose-dependent manner with similar IC50 values. In contrast, to achieve a significant reduction in proliferation by utilizing BMP2-PKD, substantially higher concentrations (~10-fold) of this ligand were required, while BMP2-P failed to completely inhibit cell proliferation. In summary, the BMP2-KD mutant appears slightly more active than wildtype BMP2 in terms of both ALP gene expression induction in ATDC5 cells as well as in inhibition of cell proliferation in KMS12-BM cells, which confirms its design as a BMP2 super-agonist.

**Fig 5:**
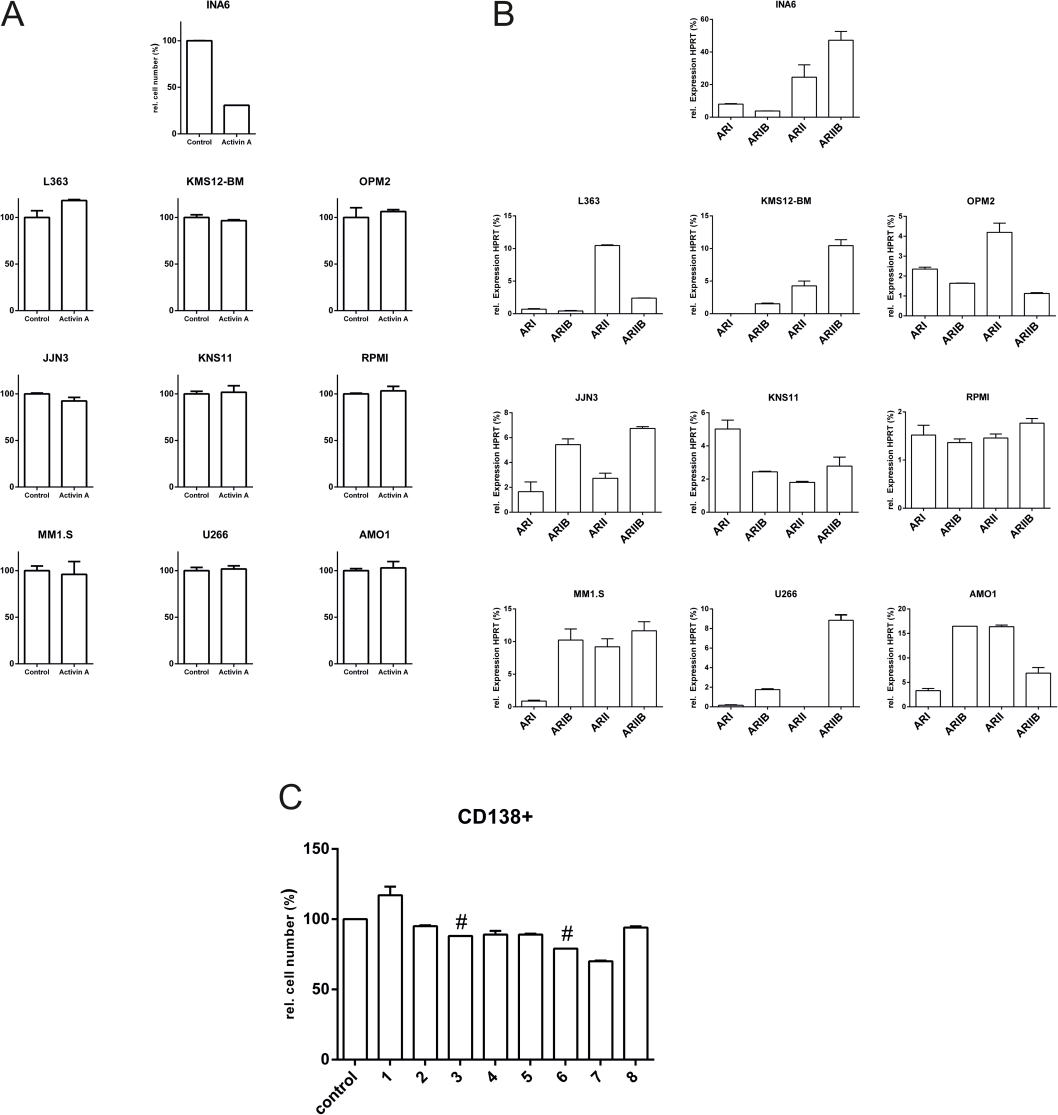
(A) A collection of different human multiple myeloma cell lines are Activin A-resistant. Ten different cell lines were incubated with or without 125 nM Activin A. After 72 h, cell proliferation was assessed by WST-1 measurements. (B) Expression profile of Activin A receptors in MM cell lines. RNA was isolated from the indicated MM cell lines, and the expression levels of the indicated receptors were determined by qRT-PCR. Expression levels were normalized to the housekeeping gene HPRT (= 100%). (C) Activin A sensitivity of primary CD138+ cells isolated from different MM patients. CD138+ cells were isolated from 8 different donors and stimulated with 125 nM Activin A for 72 h. The control cells were untreated. Cell numbers were assessed by WST-1 assay and related to the untreated cells (control). Assays were performed in duplicate (except for the values marked with a #). The figures show the mean values and standard deviation of duplicates.

The systemic elimination of Activin A by employing a receptor-based cytokine trap method was previously shown as a successful and promising strategy for the treatment of MM [6–8]. However, further studies revealed that in some cases Activin A itself represses the proliferation of certain myeloma cells [24,31]. In such cases, the removal of Activin A by a cytokine-trap approach likely exerts an adverse effect in the patient and might even promote unwanted survival and proliferation of neoplastic B-cells. To address this apparent contradiction, we analysed the Activin A sensitivity of several human myeloma cell lines (MM.1S, RPMI8226, AMO1, U266, L363, JJN3, OPM2, KMS12-BM, KMS11 and INA6), which at least in part reflect the genetic diversity of MM in terms of their impact on cell proliferation. Interestingly, only one cell line—INA6—appeared to be Activin A-sensitive (Fig 5A). The observed Activin A insensitivity might be explained by the lack of at least one of the signalling receptors (i.e., ActRIB, ActRII or ActRIIB) essential for Activin A signalling. This phenomenon was indeed observed in some cases and has been described previously [22]. We have thus analysed the presence of the Activin receptors ActRI, ActRIB, ActRII and ActRIIB on mRNA levels by qRT-PCR for all cell lines used in this study (Fig 5B). In most cases, the expression levels of the Activin receptor mRNAs were low, but detectable; however, in INA6 cells their expression levels were considerably stronger. With the presence of the necessary Activin receptors being confirmed in all MM cell lines, it seems unclear why the majority of the investigated cell lines appears to be Activin A-insensitive. Conformingly, analysis of CD138+ cells from eight MM patients also showed only a minor apoptotic effect upon Activin A stimulation (Fig 5C).

**Fig 6:**
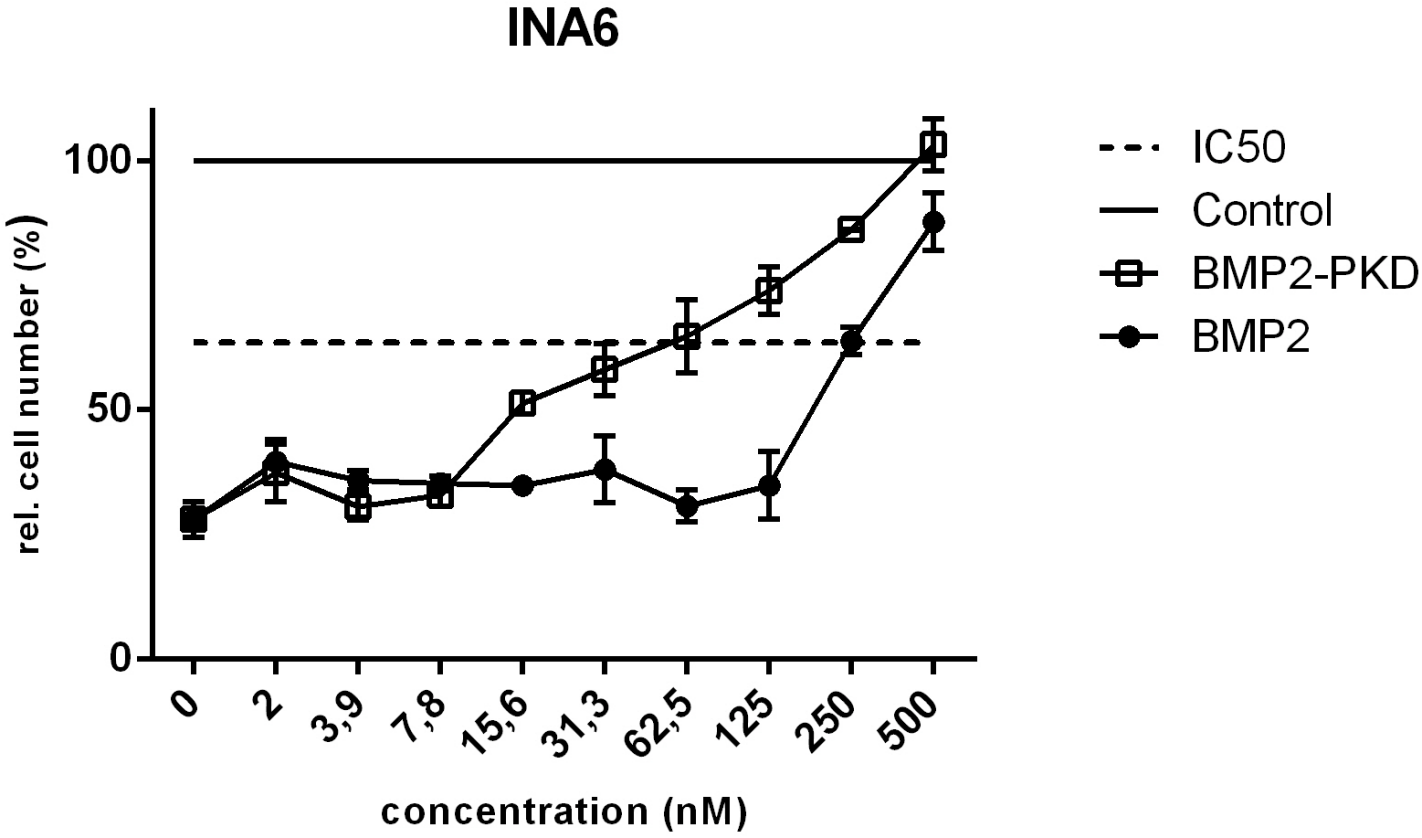
BMP2 counteracts Activin A-induced inhibition of cell proliferation in INA6 cells. INA6 cells were incubated with Activin A alone (10 nM) or co-stimulated by the addition of Activin A and increasing concentrations of either wildtype BMP2 or BMP2-PKD. Cell numbers were assessed by WST-1 measurements. The black line represents the value obtained from the untreated cells (control).The dashed line indicates the IC50 (half maximal biological inhibitor activity. In the experiment the IC50 was determined as 250 nM for BMP2 and as 62.5 nM for BMP2-PKD. The figures show the mean values of triplicates with standard deviation. The measurements were performed as three independent experiments.

The INA6 cell line, which is distinct among the cell lines investigated as Activin A-sensitive, was previously shown to be BMP4-insensitive [22]. The BMP2/4 resistance in this case might be explained by the absence of both BMP type I receptors, BMPRIA and -IB, which are essential for the initiation of BMP2-mediated signals [22]. This cell line therefore represents an excellent tool to determine whether the designed BMP2 variants counteract Activin A-mediated activities solely by the proposed competition mechanism for binding to the type II receptors, as the concomitant transduction of BMP2-specific signals via BMPRIA is impeded. Indeed, the addition of either one of the BMP2 variants dose-dependently rescued the Activin A-induced inhibition of cell proliferation in this cell line. The IC50 values determined experimentally indicate the concentration at half-maximal inhibitor activity. 250 nM wildtype BMP2 was required to obtain a 50% rescue from Activin A-mediated inhibition of cell proliferation, while the BMP2 variant having enhanced affinity for the Activin type II receptors, BMP2-PKD, 62.5 nM variant protein was required. This indicates that the variant PKD has a fourfold stronger inhibitory effect compared to wildtype BMP2. As INA6 cells lack both BMP2 type I receptors BMPRIA and -IB, which impedes the recruitment of BMP2 to the cell surface via the high-affinity type I receptors, efficacy to compete off Activin by the different BMP2 variants is solely determined by their type II receptor affinities. Thus BMP2 and BMP2-P as well as BMP2-KD and BMP2-PKD each form a pair, which have the same type II receptor binding affinity, either wildtype BMP2-like or an enhanced affinity due to carrying the KD mutation and should thus show respective similar capacities to antagonize Activin A [15,28]. A representative analysis comparing the inhibition by BMP2 and the type II receptor affinity-enhanced variant BMP2-PKD is shown in Fig 6. Our data appear to confirm the hypothesis that the counteracting biological activities of BMP2 and Activin A result from competitive binding of the two ligands to the same set of type II receptors on the cell surface.

## Discussion

In recent years, Activin A has been identified as a highly interesting target for the treatment of MM. Animal models and clinical trials have shown that systemic elimination of Activin A, e.g., via a decoy receptor, might represent a promising strategy [6–8]. In the present study, we showed that Activin A most likely exerts its inhibitory properties via a simple molecular mechanism where it competes with a distinct subset of TGFβ ligands, e.g., BMP2, for binding to an overlapping set of cell surface receptors. We showed that specifically designed BMP2 variants might be used as therapeutic agents to counteract Activin A, whose levels are elevated in MM patients [9]. A directed molecular design yielded BMP2 variants that can either act as super-agonistic BMP2 and concomitantly as a highly efficient Activin A antagonist (i.e., BMP2-KD) or, if required, exert only a super-antagonistic activity against Activin A while being otherwise inactive with regard to BMP signalling (i.e., BMP2-PKD). Particularly, the variant BMP2-KD, which exhibited increased osteogenic potential and improved Activin A-inhibiting characteristics compared to wildtype BMP2, appeared beneficial for the treatment of the major symptoms in MM. BMP2, whose endogenous biological activity is possibly reduced by Activin A overexpression and frequently observed in MM patients, is by itself a potent inducer of bone formation and regeneration. BMP2 also exerts a significant inhibitory effect on the proliferation of neoplastic B cells in MM.

Thus, the mechanism by which elevated Activin A levels lead to osteolysis and strong B-cell proliferation is most probably due to eliminating, or at least strongly reducing, the biological activities of endogenous BMPs. Because wildtype BMP2 already counteracts or attenuates Activin A activities by binding to shared type II receptors, engineering an increased binding affinity to the shared type II receptors ActRII and ActRIIB can significantly increase the inhibitory potential of the BMP2 variants (BMP2-KD and BMP2-PKD) if they are ectopically applied. If binding to BMP type I receptors is maintained by these variants, the enhanced binding affinity to the type II receptors will also result in elevated osteoanabolic capacities, which was observed for the BMP2-KD variant compared with wildtype BMP2. BMP2-KD therefore represents the most promising candidate for treatment of MM-related symptoms due to its functional gain in promoting osteoanabolic processes combined with its enhanced anti-proliferative effects on neoplastic B-cells *in vivo*. However, the therapeutic use of this mutant bears unforeseeable risks in that its systemic administration might result in non-targeted and non-specific activities on cells with mesenchymal origins [32,33]. To avoid a potential induction of ectopic bone growth in mesenchymal tissue, it might be beneficial to eliminate the osteogenic properties of the ectopically applied BMP2. To attenuate the osteogenic activity of the systemically applied BMP2, it will be necessary to reduce or even fully abrogate its SMAD1/5/8 pathway-activating capabilities. This process is best achieved using the BMP2-P variant, which, due to lacking type I receptor binding, cannot activate the SMAD1/5/8 pathway. However, despite lacking direct osteogenic activities, this variant can still compete with Activin A for binding to the type II receptors ActRII and ActRIIB and therefore antagonize Activin A activities. Moreover, although the BMP2-P variant cannot bind to BMP type I receptors, it can still bind the BMP antagonist Noggin similar to wildtype BMP2 [29,34]. Noggin is often co-expressed with BMPs to dampen BMP activity via a negative feedback loop. Because the BMP2-P variant can neutralize Noggin and thereby release endogenous BMPs from complexes with Noggin, it may therefore indirectly increase osteogenic activities at sites where BMPs are endogenously produced [29,34,35]. The antagonistic and site-specific osteogenic potential of this variant has already been shown in cell culture as well as in a rat model and might be superior in many aspects to wildtype BMP2 administration [29,34].

The same features, but provided with enhanced Activin A-competing properties, are realized with the BMP2-PKD variant. This variant combines increased binding affinity for Activin type II receptors and can therefore inhibit Activin A activities more efficiently. This feature might be beneficial in clinical use in terms of safety because lower doses of the systemically applied BMP2 variant can be administered. However, what would be the risks of systemically applying BMP2 proteins with preserved intrinsic osteogenic activities, such as wild type BMP2 or BMP2-KD? Basically, there is no clear answer to this question due to the lack of clinical studies and animal models. There might be a risk to force ectopic bone formation in mesenchymal tissue or to promote certain cancer activities, especially because it has been previously proposed that BMPs might be involved in mesenchymal stem cell (MSC) differentiation and in processes associated with cancer, such as epithelial to mesenchymal transition (EMT) [36]. However, BMP levels in the blood of healthy people as well as in MM patients are very low, which does not allow a conclusion in terms of appropriate doses for BMP2 application in such a scenario [37,38]. Furthermore, Activin A levels in the blood also appear to be very low (400–1500 pg/ml) [9]; but only subnanomolar concentrations are required to initiate Activin A activity like BMP inhibition [39]. In contrast, BMPs require concentrations in the range of 10 to 50 nM to exert their osteogenic activities [40]. Therefore, to exclusively inhibit Activin A-specific activities without inducing specific BMP-related responses, rather low concentrations of a BMP-derived Activin A antagonist should be applied. This situation suggests that the Activin A super-antagonist BMP2-PKD is likely the best option for such a treatment regimen.

Still, there are two more facts that need to be discussed. First, neoplastic cells do not appear to be Activin A-insensitive in all MM patients. In these cases, Activin A exerts an anti-proliferative effect similar to BMP2 and our generated BMP variants. Thus, BMP2 administration can potentially provoke unwanted side effects through the inhibition of endogenous Activin A in these cases [24]. In contrast, only one (INA6) out of ten MM cell lines tested responded to Activin A. Furthermore, analysis of the CD138+ cells from eight MM patients showed only minor apoptotic effects upon Activin A stimulation. Last but not least, the increased Activin A levels in MM patients correlate with extensive bone involvement and inferior survival [9]. Equally diverse directed antagonists against endogenous Activin A that act in the form of decoy receptors have also displayed a positive effect on MM [6–8,31,41,42]. For these reasons, the advantages for blocking Activin A via BMPs predominate on the receptor level.

Furthermore, Olsen and co-workers have already shown that Activin A can antagonize BMP6 and BMP9 by competing with binding to the two type II receptors ActRII and ActRIIB in MM cells [39]. Interestingly there seem to be substantial differences when comparing their results with our data. First, very low concentrations of Activin A were needed to antagonize BMP6 and BMP9. Second, the activities of BMP2 and BMP4 could not be antagonized by similar levels of Activin A. However these results seemingly contradicting our data appear only different at first glance. Instead we consider differences in the binding kinetics and the formation of the signaling-active ligand-receptor complex between BMP2 and BMP6/BMP9 to account for this discrepancy.

To explain this oppositional observations we would like to examine the binding characteristics of BMP2 from two different perspectives. Firstly Activin A can act as an BMP2 inhibitor in the cell lines C2C12 and KMS12-BM as shown in this paper (Fig 2A and 2B). But in these cells, Activin A concentrations several times higher than the BMP2 concentration used for stimulation (10 fold and more) were necessary for effective inhibition of BMP2-mediated effects. In order to act as efficient inhibitor in this scenario, Activin A must prevent the formation of an active BMP signaling complex, which (in these cells) comprises of two type I receptors, two type II receptors and the BMP2 dimer. The formation of such an activating ligand-receptor complex is considered to occur as a two step-reaction though, since the affinity of the BMP2 ligand for the type I and II receptors are dramatically different. In vitro measurements have shown that the affinity of BMP2 for its type II receptors is about 10 to 60 times lower than for its type I receptors [14]. Therefore in the first step BMP2 binds to a type I receptor (BMPRIA or -IB) with high affinity and subsequently this membrane-located binary complex then recruits the “low-affinity” type II receptors into the complex to initiate downstream signaling. But defining the type II receptors ActRII, ActRIIB and BMPRII as being low-affinity receptors of BMP2 might be misleading since strictly speaking the low affinity only holds true for the scenario where BMP2 directly binds from the extracellular space to its type II receptors. If, however, BMP2 has been already recruited to the membrane via binding first to its type I receptors, subsequent interaction with the type II receptors will occur in two-dimensional space, i.e. the binary complex of BMP2-type I receptor will recruit the type II receptor in the membrane and thus this interaction is limited to a lateral search/interaction. Various theoretical analyses clearly showed that this reduction in the degrees of freedom by changing a three-dimensional to a two-dimensional search can significantly enhance reaction rates of the second interaction step occuring within the membrane [43–46]. This suggests that we will observe a higher apparent affinity for the type II receptors for the BMP ligand when the interaction occurs within the membrane as is typically found by in vitro setups measuring 3D interactions such as surface plasmon resonance or calorimetry [26,47]. Thus despite on paper Activin A binds the type II receptors with higher affinities than BMP2, the type I receptor-facilitated interaction of BMP2 with the type II receptors within the membrane will make Activin A a rather weak inhibitor for BMP2 requiring unexpected high concentrations of Activin A. To outcompete BMP2, almost all type II receptors must be occupied by Activin A, as otherwise the binary membrane-located BMP2/type I receptor complex will efficiently recruit its type II receptor (referred to as scenario A).

The scenario will get different when the blocking of Activin A by BMP2 in INA6 cells is analyzed (referred to as scenario B). Although this might be considered the reverse situation as described above, here, a much higher BMP2 concentration is required to antagonize the effects induced by a quite low nanomolar concentration of Activin A (see Fig 6). The difference is due to the fact that INA6 cells do not express the high-affinity type I receptors required for BMP2, i.e. BMPRIA and -IB), BMP2 mediated blockage of Activin A type II receptor binding will not be facilitated by a first recruitment of BMP2 to the cell membrane. Thus Activin A and BMP2 will compete in directly binding to the shared type II receptors hence employing the affinities as were determined by in vitro interaction methodologies such as surface plasmon resonance or calorimetry. Since BMP2 exhibits affinities to its three type II receptors which are about 15 to 35 times lower as found for Activin A [14], much higher BMP2 concentrations are needed to impede Activin A receptor binding and activation. This mechanism is consistent with the BMP2 variant PKD, which binds the Activin type II receptors with almost Activin-A like affinities but has a very low affinity for BMP type I receptors, exhibiting a higher antagonistic capacity against Activin A (see Fig 6). Although the mechanism underlying the mutual antagonism of Activin A and BMPs is due to a direct competition for binding to a set of shared type II receptors, the particular expression profile of BMP type I and type II receptors in the cells addressed as well as the receptor binding profile of the respective BMP will strongly affect the inhibitory efficacy either of Activin A or that of the BMP.

With these considerations we might be able to understand why Olsen et al. required only low concentrations of Activin A to inhibit BMP9 and BMP6-mediated inhibition of cell proliferation in INA6 and IH-1 myeloma cell lines, but could not detect any effect of similar levels of Activin A on BMP2/4 in IH-1 cells [39]. BMP6 does not bind any TGFβ type I receptors with high affinity, but has intermediate affinity for Activin type II receptors and can thus be antagonized by Activin efficiently employing by competing for binding to type II receptors directly from the extracellular space (like scenario B). In contrast BMP9 binds with high affinity to the type I receptor ALK1, which is however absent in MM cells and thus shows a similar antagonistic profile against Activin A as observed for BMP6 with the only difference that BMP9 also binds type II receptors with higher affinity than BMP6. The failure of BMP2 and -4 to antagonize Activin A in the MM cell line IH-1 observed in the study of Olsen et al. is due to the fact that IH-1 cells express all three type I receptors [22] bound by BMP2 and -4 and thus both TGFβ ligands must be competed when already being recruited to the cell surface by their high-affinity type I receptors (see scenario A above), which however as shown by our data requires much higher concentrations of Activin A as have been used in the study of Olsen et al. [39].

One further point is still of fundamental interest. Why did we use BMP2 and not a different BMP ligand? BMP2 and the variants used in this study can potentially inhibit BMP6, BMP9, and various other BMPs due to shared use of the three type II receptors ActRII, ActRIIB and BMPRII. This appears to be counterproductive, as BMP6 can more strongly induce apoptosis in primary myeloma cells via the type I receptor ACVRI (ALK2) as BMP2 and -4 can do via the BMPRIA and/or -IB [23,48]. Furthermore, ACVR1/ALK2 is -in contrast to BMPRIA and -IB- ubiquitously expressed in myeloma cells, which potentially renders BMP6 a more versatile Activin A antagonist than BMP2 [49]. We have employed BMP2 in our studies for two main reasons. Firstly, we show that also BMP2 can act as an Activin A antagonist in MM. Secondly, BMP2 and particularly its variants were used as model to demonstrate that BMP molecules with custom-tailored functions can be generated by molecular design for a specific use in MM therapy. We think that the functionalities of these BMP2 variants could also be transfered to BMP6, which potentially would make BMP6 an even more versatile Activin A antagonist. However in order to gain further insights into the therapeutic potential of this panel of BMP2 and possible BMP6 variants, testing in animal models is required to determine the functions of such Activin A antagonists at Activin A sources and sinks. Even though the applicability of such BMP-based Activin A antagonists is yet to be shown in animal testing, we think that these BMP2 variants nevertheless present a powerful tool for the treatment of MM in patients by gradually affecting Activin A-mediated activities and thereby mediating significant improvements in the clinical symptoms associated with this disease.

## Conclusions

In this work, we show that Activin A directly inhibits BMP2-induced anti-proliferative activities in MM cell lines. In addition, custom-designed BMP2 variants with engineered tailored receptor binding affinities for type II and type I receptors can be used as superior Activin A antagonists simultaneously serving as BMP2 super-agonists to potentially overcome bone erosions observed in MM. These features might be clinically exploited as a new therapeutic option for the treatment of MM.
